# Investigating the properties of TBA variants with twin thrombin binding domains

**DOI:** 10.1038/s41598-019-45526-z

**Published:** 2019-06-24

**Authors:** Teresa Amato, Antonella Virgilio, Luciano Pirone, Valentina Vellecco, Mariarosaria Bucci, Emilia Pedone, Veronica Esposito, Aldo Galeone

**Affiliations:** 1 Dipartimento di Farmacia, Università degli Studi di Napoli Federico II, Via D. Montesano 49, 80131 Napoli, Italy; 2 Istituto di Biostrutture e Bioimmagini, CNR, Via Mezzocannone 16, 80134 Napoli, Italy

**Keywords:** DNA, Molecular biology

## Abstract

In this paper, we report studies concerning thrombin binding aptamer (TBA) dimeric derivatives in which the 3′-ends of two TBA sequences have been joined by means of linkers containing adenosine or thymidine residues and/or a glycerol moiety. CD and electrophoretic investigations indicate that all modified aptamers are able to form G-quadruplex domains resembling that of the parent TBA structure. However, isothermal titration calorimetry measurements of the aptamer/thrombin interaction point to different affinities to the target protein, depending on the type of linker. Consistently, the best ligands for thrombin show anticoagulant activities higher than TBA. Interestingly, two dimeric aptamers with the most promising properties also show far higher resistances in biological environment than TBA.

## Introduction

**Figure 1:**
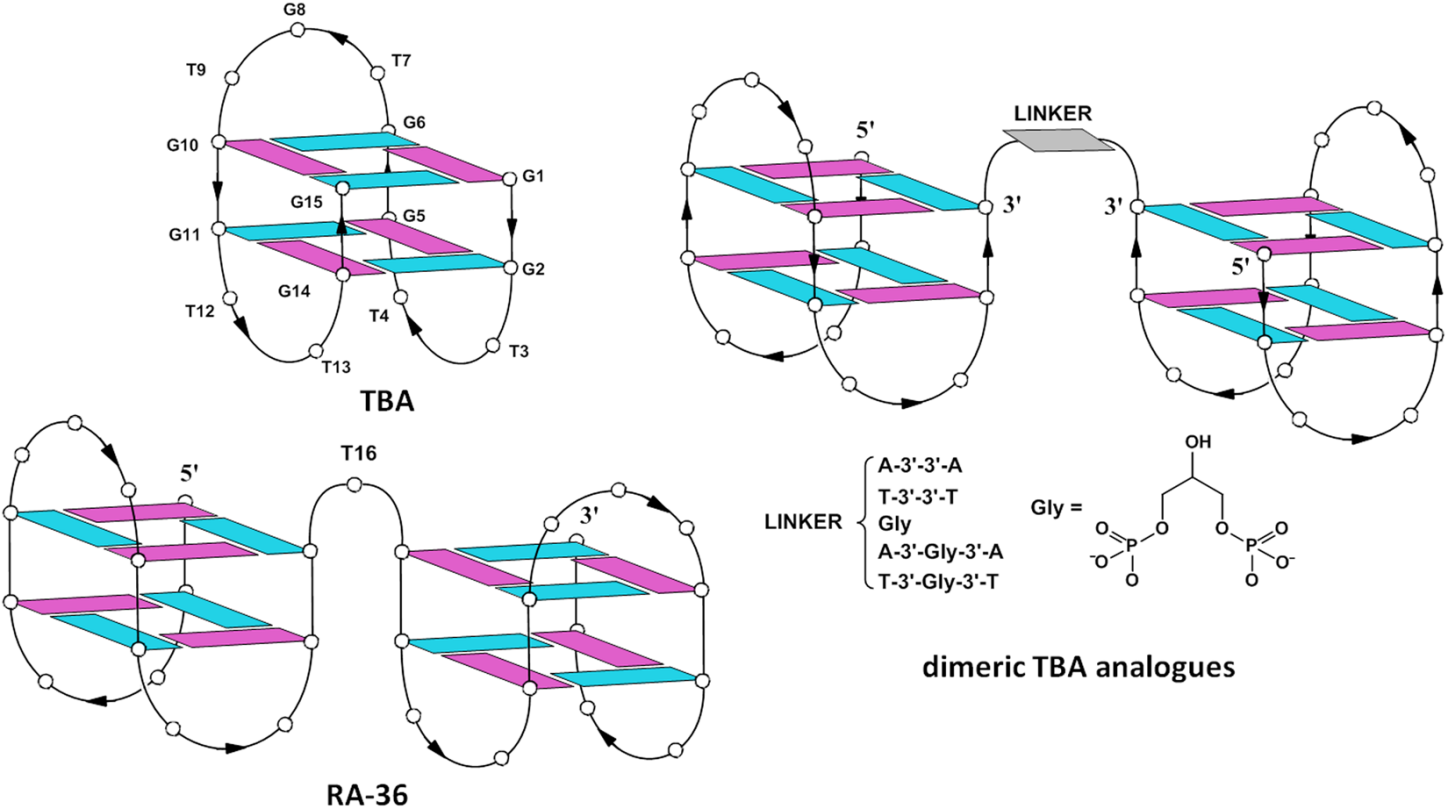
G-quadruplex structures formed by TBA, RA-36 and the new dimeric TBA analogues. Guanosines adopting *anti* and *syn* glycosidic conformations are indicated in light blue and purple, respectively. For clarity, the loop residues are indicated only by white circles.

Nucleic acid aptamers are DNA or RNA ligands characterized by high affinity and specificity to their target molecules, which can be both proteins, and small molecules. Due to their favorable properties, aptamers can find applications in many fields including diagnostics and therapeutics^1^. Although these ligands can be discovered by different strategies, in general, they are selected through combinatorial techniques, overall called SELEX^2^. However, a DNA or RNA ligand molecule discovered by SELEX techniques can be rarely used unmodified, mainly because of the poor resistance of natural oligonucleotides in biological environments. For these reasons, the original aptamer sequence is often subject to post-SELEX modifications aimed at improving their general properties such as, affinity and specificity toward the target, thermal stability, and resistance to ubiquitous endo- and exonucleases^3^. The most common chemical modifications include introduction of modified bases, sugar-phosphate backbone alteration and conjugation with other molecules. A minor number of researches have been devoted to aptamer dimerization or multivalent aptamer construction^4^ which consist in the design of ligands with two or more binding domains to the target, although these structural modifications have been proven to provide the new construct ligands with better properties than the original aptamer. For example, in 2008 Hasegawa *et al*. described a dimeric anti-VEGF (vascular endothelial growth factor) aptamer showing a lower dissociation constant value than that of the parent monomer^5^. In a previous research, multivalent circular aptamers against thrombin were designed and assembled, displaying improved anticoagulant potencies with EC_50_ values better than that of the canonical GS-522 thrombin DNA aptamer^6^. Two further anticoagulant aptamers, namely the G-quadruplex thrombin binding aptamer (TBA, also called HD1, Fig. 1 and Table 1), and HD22, were subject to dimerization through the formation of a stable poly-A/poly-T duplex joining their binding domains by hybridization (aptabody)^7,8^. TBA represents also the precursor of one of the most simple and interesting dimeric aptamer, namely the 31-mer RA-36 (Fig. 1 and Table 1), in which two TBA pharmacophore modules are connected through a thymidine residue. The properties of RA-36 have been extensively investigated in comparison with other anti-thrombin aptamers^9–13^. Collected data have shown that RA-36 is an efficient thrombin inhibitor with a dose-dependent effect. Furthermore, animal tests have suggested a species-specificity of RA-36^9,10^. In a further study the antithrombotic activity of RA-36 has been evaluated by a murine thrombosis model^12^. Moreover, its pharmacodynamic and pharmacokinetic properties have been widely evaluated^13^. Interestingly, truncation studies have shown that the two G-quadruplex domains are not functionally equivalent, having the 5′-G-quadruplex module an inhibitory activity higher than the precursor TBA and the 3′-G-quadruplex module^11^.

**Table 1:** Names, sequences and melting temperatures (T_m_s) of the ODNs investigated. See Methods section for details. Gly is here the abbreviation for glycerol.

Name	Sequence	T_m_ (°C) ± 1
TBA	5′-GGTTGGTGTGGTTGG-3′	33
RA-36	5′-TBA-T-TBA-3′	32
AA	5′-TBA-A-3′-3′-A-TBA-5′	37
TT	5′-TBA-T-3′-3′-T-TBA-5′	33
Gly	5′-TBA-3′-Gly-3′-TBA-5′	35
AGlyA	5′-TBA-A-3′-Gly-3′-A-TBA-5′	35
TGlyT	5′-TBA-T-3′-Gly-3′-T-TBA-5′	29

The above described findings have inspired us the design of some dimeric TBA derivatives in which two 5′-G-quadruplex TBA domains are connected through their 3′-ends by symmetric linkers composed by adenosine or thymidine residues and/or a glycerol (abbreviated as Gly) moiety (Figs 1, S1 and Table 1). The design of these dimeric aptamers shows the following advantages respect to other anti-thrombin dimeric aptamers: (1) they are characterized by the two most active 5′-G-quadruplex domains and, (2) thanks to the inversion of polarity, they do not possess free 3′-ends that are susceptible to degradation by the most biologically abundant 3′-exonucleases. Although the introduction of inversion of polarity sites in G-quadruplex structures was proposed some years ago^14–16^, only recently this strategy has been successfully used as post-SELEX modification to increase the biological stability of TBA^17,18^ or to modulate its biological activities^15,16^. In this report, an inversion of polarity site has been exploited to join two G-quadruplex TBA domains. Since TBA has been proven to interact with the fibrinogen-recognition exosite (EXO I), this one is most likely the target also in the case of the TBA variants here described. The new dimeric aptamers have been investigated by circular dichroism (CD) and polyacrylamide gel electrophoresis (PAGE). Moreover, their abilities to bind thrombin have been evaluated by isothermal titration calorimetry (ITC), while the anticoagulation activity has been tested by the prothrombin time (PT) assay. Finally, the resistance in biological environments has been estimated through a fetal bovine serum (FBS) stability assay.

The whole of the data have indicated that all the dimeric aptamers are able to form G-quadruplex structures, although the affinity to thrombin depends on the type of linker. Remarkably, most of the dimeric TBA derivatives have shown higher anticoagulant activities than those of both TBA and RA-36. Furthermore, all new aptamers have shown higher nuclease resistance than TBA and, in some cases, also than RA-36.

## Results and Discussion

### Structural insight into the dimeric TBA derivatives

In order to obtain preliminary structural information about the conformation adopted by the dimeric TBA derivatives and evaluate the effects of the symmetric linkers or the inversion of polarity site, CD spectra and CD melting and annealing experiments were acquired for all derivatives and compared with the data of their natural counterpart TBA.

**Figure 2:**
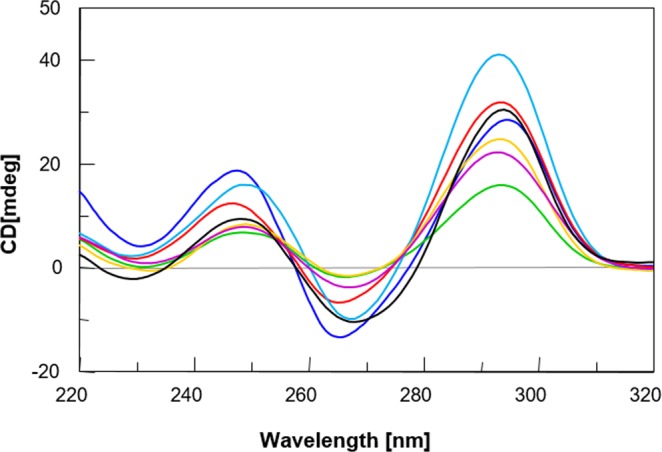
CD spectra of the investigated ODNs. **AA** (dark blue), **TT** (purple), **Gly** (red), **TGlyT** (yellow) and **AGlyA** (green). TBA (black) and RA-36 (light blue) ODNs have been introduced as references. See the main text and the Methods section for details.

The CD spectrum of the TBA is characterized by two positive bands at 247 and 295 nm and a negative one at 266 nm. This profile is typical of an antiparallel G-quadruplex structure in which *anti* and *syn* guanosines alternate both along the strands and in each G-tetrad. Considering the distinctiveness of this profile, a close comparison between CD spectra of the dimeric TBA derivatives and that of their natural counterpart can be considered a useful method to forthrightly estimate the effects of these modifications on the G-quadruplex structure. In particular, besides some differences concerning the band intensities and negligible dissimilarities relating their wavelength maxima, the CD spectra of all dimeric derivatives and TBA, (Fig. 2) performed at 10 °C, are quite similar, thus exhibiting analogous positive and negative bands, indicative of the formation of antiparallel G-quadruplex structures comparable to that of the unmodified aptamer TBA for all the dimeric derivatives.

**Figure 3:**
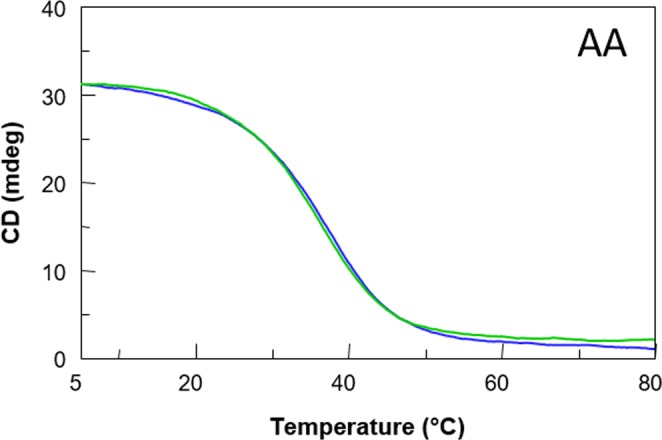
Representative CD melting (blue) and annealing (green) curves of the G-quadruplex formed by **AA**. See the main text and the Methods section for details.

CD melting and annealing measurements have also been used to evaluate the thermal stability of the modified aptamers (Figs 3 and S2). The well-defined sigmoidal CD melting and annealing profiles of the modified TBA dimers have confidently allowed us the measurement of the melting temperatures (T_m_s) (Table 1). A comparison of all data shows that while the T_m_s of **TT**, **Gly**, **AGlyA** and RA-36 can be considered similar to that of the unmodified aptamer, taking into account the experimental error, **AA** is characterized by a slightly higher T_m_ (ΔT = + 4 °C) than the natural counterpart. On the other hand, **TGlyT** showed a slightly lower T_m_ (ΔT = −4 °C) than TBA. The clear absence of hysteresis between heating and cooling profiles in all cases, indicating fast equilibrium kinetics of the system in the experimental conditions, would suggest the occurrence of a monomolecular G-quadruplex structure.

The ODNs investigated were further analyzed by PAGE and compared with TBA and RA-36 used as references (Fig. S3). ODNs **TT**, **Gly**, and **TGlyT** have showed bands with electrophoretic motilities rather similar to that of the dimeric TBA derivative RA-36 and quite slower than TBA, thus suggesting that the dimeric G-quadruplexes do not form higher ordered structures. Little differences concerning their motilities could be ascribed to the effect on the migration rate of the different linkers connecting the twin TBA domains. However, in the case of ODNs **AA** and **AGlyA** two bands are present, being the minor slow-migrating one characterized by a similar motility as RA-36. Since their CD profiles are comparable to that of TBA (Fig. 2) and both their anticoagulant activity and affinity to thrombin were preserved (see below), it is reasonable to hypothesize that this electrophoretic behaviour does not involve the ability of **AA** and **AGlyA** to adopt independent twin “chair-like” G-quadruplex structures. Taking into account that both these ODNs have adenosine residues in their linkers, the presence of the fast-migrating band could be tentatively explained with the propensity of the purine bases to interact with the adjacent G-quadruplex, thus forming prevalent more compact structures. In the cases of **Gly**, **AGlyA** and **TGlyT** a further minor band with an electrophoretic motility comparable to that of TBA is scarcely noticeable. This result would suggest a higher lability of the Gly-containing linkers that caused a slight degradation in monomers of the ODN samples used for the electrophoresis. The evaluation of the resistance of the dimeric TBA derivatives by FBS assay (see below) is clearly in agreement with this hypothesis.

### Evaluation of the aptamer/thrombin interactions

**Figure 4:**
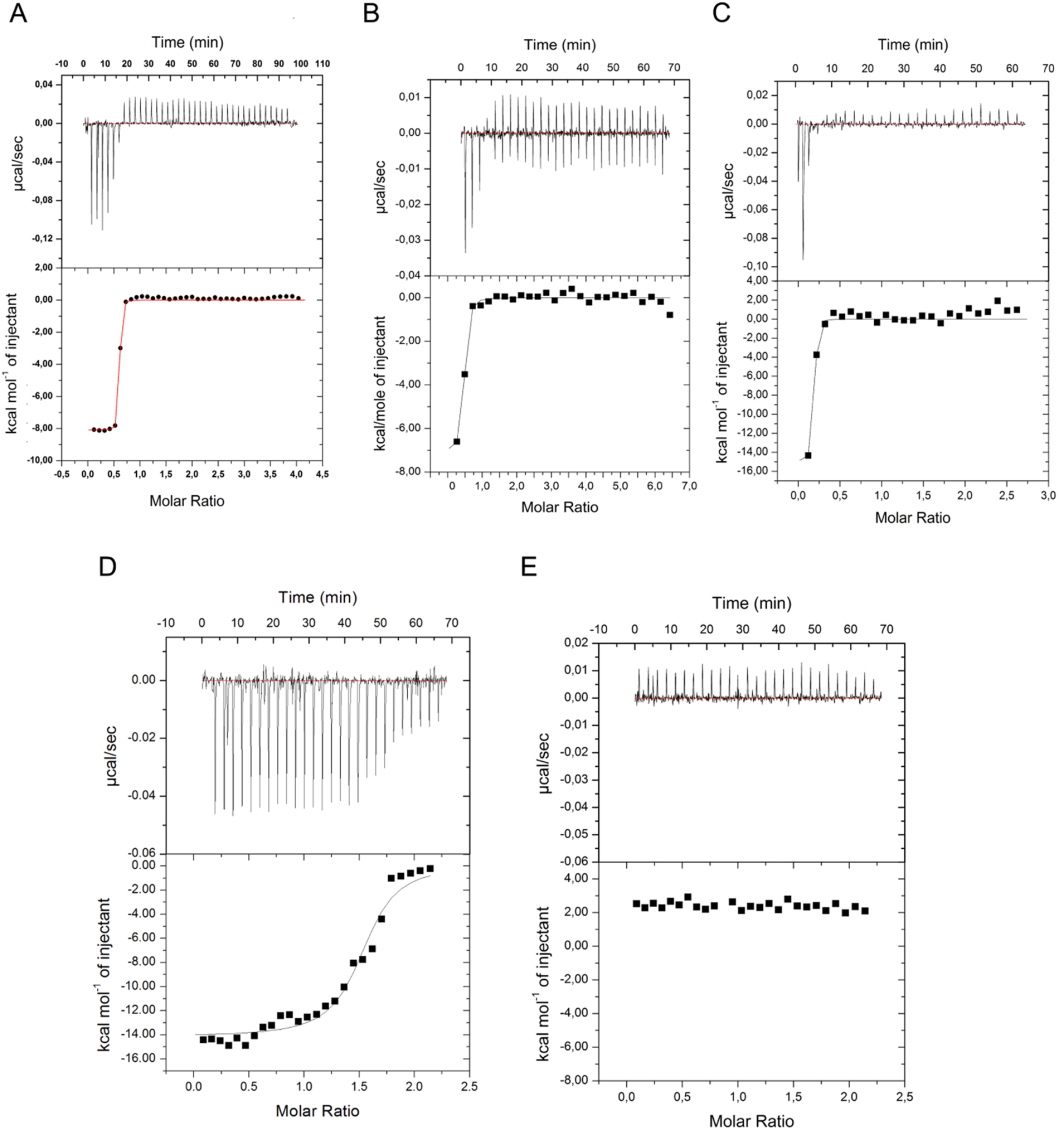
Isothermal titration calorimetry data for the binding of thrombin with the various ODNs. (**A**) TBA at 100 μM *versus* 10 μM thrombin; (**B**) TBA, (**C**) TT and (**D**) RA-36 each at 25 μM *versus* 2.5 μM thrombin. (**E**) Control: TBA at 25 μM *versus* buffer. The top and bottom panels report raw and integrated data, respectively.

In order to assess and quantify the interaction between TBA or its dimeric analogues and human α-thrombin, ITC measurements were performed. Firstly, the interaction between TBA and the thrombin was analysed at two different protein concentrations to get a more reliable K_d_ value. In both cases, the evolution of the heat exchange during the titration is indicative of a tight binding between the two molecules (Fig. 4A,B). ITC data, after integration and correction of heats for dilution, were fitted with the one set of binding sites model. The optimal fitting of the experimental data provided a reliable dissociation constant K_d_ of 8 ± 1 nM. Secondly, all the other ODNs have been studied considering the concentration value used in the biological assays. All the TBA dimeric analogs have shown to be able to bind thrombin, and in detail ODNs **AA**, **TT** (Fig. 4C) and **AGlyA** show comparable K_d_ values with TBA (K_d_ = 8 nM), namely 10, 5 and 5 nM, respectively (Fig. S4, Table S1) while RA-36 displays the lowest affinity (K_d_ = 100 nM) (Fig. 4D). Interestingly, ODNs **AA** and **TT** present a trend ascribable to an exothermic reaction differently from the **Gly**, **AGlyA** and **TGlyT** variants showing an endothermic titration profile. Such behavior suggests that the linkers containing a glycerol moiety could be involved in the interaction with the thrombin.

### Anticoagulant activity

**Figure 5:**
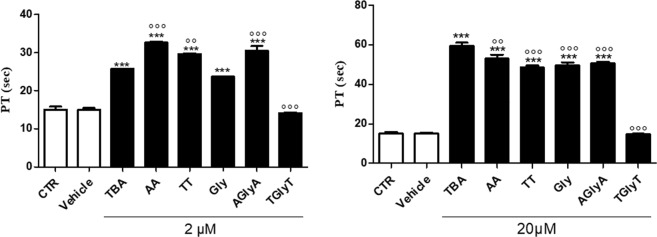
PT values of the dimeric TBA analogues and their natural counterpart at 2 and 20 μM and 15 min incubation. See Methods section for details. ***p < 0.001 vs. vehicle, °°p < 0.01, °°°p < 0.001 vs. TBA (n = 3).

In order to evaluate the anticoagulant properties of the TBA dimeric analogs, the ODNs have been subjected to PT assay and their anticoagulant activities have been compared to TBA (Fig. 5). The results show that all the aptamers, except **TGlyT**, display a prolonged PT time at both concentration tested (2 and 20 µM). However, some differences among the ODNs tested have been found, when compared to TBA. In details, at the lowest concentration used i.e 2 µM, the PT detected for **TT**, **AglyA** and **AA** has been 29.6 ± 0.18, 30.5 ± 1.2 and 32.6 ± 0.32 sec, respectively. These PT values are significantly higher when compared to their natural counterpart TBA (25.7 ± 0.10 sec) and to RA-36 for which a PT value of about 23 sec has been reported^12^. Otherwise, evaluation of PT in plasma incubated with **Gly** reveals an increased PT value superimposable to that observed for TBA. These data are quite in agreement with those regarding the dissociation constant values of the complex aptamer/thrombin calculated by ITC (Table S1). Concerning the absence of anticoagulant activity of **TGlyT**, it should be noted that this variant shows both the lowest thermal stability (Table 1) and the highest dissociation constant (Table S1), thus clearly indicating that these two negative characteristics contribute in collapsing the biological activity. Unexpectedly, when the TBA dimeric analogues have been evaluated at the highest concentration (20 µM), a reduction of the anticoagulant properties has been detected, compared to TBA. These data suggest a negative effect of the concentration that could be due to an increase of the intermolecular interactions among the G-quadruplex domains in these conditions.

### Resistance in biological environments

**Figure 6:**
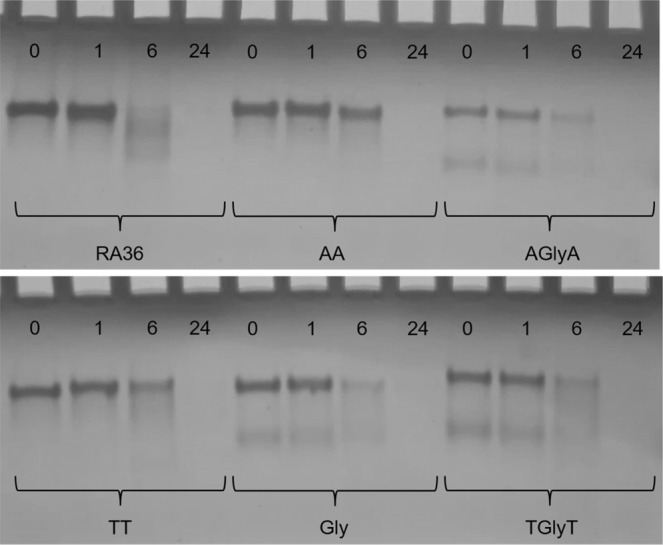
Stability of the investigated ODNs in 10% fetal bovine serum (FBS), as monitored by non-denaturing PAGE. Time points 0, 1, 6 and 24 are in hours. See the Methods section for experimental details.

In order to test the resistance in biological environments, all the TBA dimeric analogues were undergone to a degradation assay in fetal bovine serum (Fig. 6) and analyzed by gel electrophoresis. Data indicated that there are still significant amounts of undamaged ODNs **AA** and **TT** after 6 h. On the contrary, an almost complete degradation is evident for RA-36 after the same time while TBA has been proven to degrade totally after only 1 h in the same conditions^19^. These data can be explained taking into account that **AA** and **TT** are characterized by only free 5′-ends while TBA and RA-36 have canonical sequences with a 3′-end more sensitive to nucleases. Unexpectedly, **Gly**, **AGlyA** and **TGlyT** have shown a minor resistance after 6 h than the other TBA dimer analogues with an inversion of polarity, although they also exhibit only 5′-ends. The data suggest that the linkers containing a glycerol moiety make the molecule less resistant in biological environments.

## Methods

### Oligonucleotides synthesis and purification

The oligonucleotides in Table 1 were synthesized on a Millipore Cyclone Plus DNA synthesizer using solid phase β-cyanoethyl phosphoramidite chemistry at 15 µmol scale. For all ODNs an universal support was used. The synthesis of the 3′-5′ tracts was performed by using normal 3′-phosphoramidites, whereas the 5′-3′ tracts were synthesized by using 5′-phosphoramidites. For the synthesis of modified oligonucleotides containing the glycerol linker, a non-nucleosidic phosphoramidite building block, synthesized as described in the supplementary material, was coupled to universal solid support before using normal 3′-phosphoramidites (Fig. S1). The oligomers were detached from the support and deprotected by treatment with concentrated aqueous ammonia at 80 °C overnight. The combined filtrates and washings were concentrated under reduced pressure, redissolved in H_2_O, analyzed and purified by high-performance liquid chromatography on a Nucleogel SAX column (Macherey–Nagel, 1000-8/46), using buffer A: 20 mM NaH_2_PO_4_/Na_2_HPO_4_ aqueous solution (pH 7.0) containing 20% (v/v) CH_3_CN and buffer B: 1 M NaCl, 20 mM NaH_2_PO_4_/Na_2_HPO_4_ aqueous solution (pH 7.0) containing 20% (v/v) CH_3_CN; a linear gradient from 0 to 100% B in 45 min and a flow rate of 1 ml/min were used. The fractions of the oligomers were collected and successively desalted by Sep-pak cartridges (C-18). The isolated oligomers proved to be >98% pure by NMR (Fig. S5).

### Circular dichroism spectroscopy

CD samples of modified oligonucleotides and their natural counterpart were prepared at ODN concentration of 50 µM by using PBS (Sigma-Aldrich; 10 mM phosphate buffer, 2.7 mM KCl, 137 mM NaCl, pH 7.4) and subjected to the annealing procedure (heating at 90 °C and quickly cooling at 4 °C). CD spectra of all quadruplexes and CD melting/annealing curves were registered on a Jasco 715 CD spectropolarimeter. For the CD spectra, the wavelength was varied from 220 to 320 nm at 100 nm min^−1^ scan rate, and the spectra recorded with a response of 16 s, at 2.0 nm bandwidth and normalized by subtraction of the background scan with buffer. The temperature was kept constant at 5 °C with a thermoelectrically controlled cell holder (Jasco PTC-348). CD melting/annealing curves were registered as a function of temperature (range: 5–80 °C) for all quadruplexes at their maximum Cotton effect wavelengths. The CD data were recorded in a 0.1 cm pathlength cuvette with a scan rate of 0.5 °C/min.

### Polyacrylamide gel electrophoresis

All oligonucleotides were analyzed by non-denaturing PAGE. Samples annealed in PBS were loaded on a 20% polyacrylamide gel containing Tris–Borate-EDTA (TBE) 2.5x and NaCl 50 mM. The run buffer was TBE 1x containing 100 mM NaCl. For all samples, a solution of glycerol/TBE 1x-100 mM NaCl 2:1 was added just before loading. Electrophoresis was performed at 8 V/cm at a temperature close to 10 °C. Bands were visualized by UV shadowing.

### Isothermal titration calorimetry

ITC studies were performed at 22 °C with an ITC200 calorimeter (MicroCal/GE Healthcare). Before all titration experiments the protein was dialyzed against PBS pH 7.4 and the ODNs were resuspended in the same buffer. TBA (25 or 100 μM) and its dimeric analogues **AA**, **TT**, **Gly**, **AGlyA**, **TGlyT** and RA-36 (25 μM) were titrated into a solution of human α-thrombin (2.5 μM or 10 μM) (Hematologic Technologies Inc., HCT-0020)^20^. To exclude the presence of artifacts due to ligand dilution into the protein buffer, ITC measurements were performed by titration of ODNs into the buffer. All data were analyzed and fitted using the Microcal Origin version 7.0 software package. Dissociation constants were determined by fitting the data using a one-set-of-site-binding model. ITC runs were repeated twice to evaluate the reproducibility of the results.

### Prothrombin time (PT) assay

PT assay was performed on human plasma accordingly to previous investigations^21–23^. Briefly this method relies on the high sensitivity of thromboplastin reagent based on recombinant human tissue factors. The addition of recombiplastin to the plasma, in presence of calcium ions, activates the extrinsic pathway that culminates with the conversion of fibrinogen into fibrin and therefore with a formation of a solid gel. In our experimental conditions, TBA and the modified aptamers were tested at two different concentration: 2 µM and 20 µM. All the ODNs or vehicle (phosphate buffer saline, PBS) were incubated with 100 μl of plasma at 37 °C for 15 min and then 200 μl of the kit solution containing recombiplastin was added with the consequent activation of the extrinsic pathway. The PT measurement was produced in triplicate and the average and the standard error values were calculated and expressed as seconds. The basal clotting time was evaluated by measuring the clotting time in presence of vehicle.

### Fetal bovine serum assay

Nuclease stability assay of modified oligonucleotides was conducted in 10% fetal bovine serum (FBS) diluted with Dulbecco’s Modified Eagle’s Medium (DMEM) at 37 °C. Approximately 15 nmol of stock solution of each ODN (~3 O.D.U.) was evaporated to dryness under reduced pressure and then, incubated with 500 μl 10% FBS at 37 °C. At 0, 1, 6 and 24 h, 125 μl of samples were collected and stored at –20 °C for at least 20 min. The samples were evaporated to dryness and then 10 μl of gel loading buffer and 10 μl of autoclaved water was added. 10 μl of the mixture was used for polyacrylamide gel electrophoresis (PAGE), which was carried out at room temperature using 20% polyacrylamide gel in 1 × TBE buffer (Tris-borate-EDTA). The degradation patterns on the gel were visualized by UV shadowing.

## Conclusion

The dimerization of the active domain is one of the strategies often used to improve the properties of the aptamers. Just like others aptamers, also the TBA, being one of the most investigated aptamers, has been the subject of dimerization studies. In particular, the properties of a dimer TBA analogue containing two domains separated by a thymidine residue (namely, the 31-mer RA-36) have been extensively investigated. However, since RA-36 is a canonical DNA sequence, as the TBA, is easily degraded in biological environments. Furthermore, truncation studies have proven that the 5′-G-quadruplex domain of RA-36 contributes to the anticoagulant activity in a higher extent than the 3′-G-quadruplex domain. In this frame, we have synthesized five TBA dimeric analogues in which two 5′-G-quadruplex domains are connected by their 3′-ends using linkers containing two nucleotides and/or a glycerol moiety. CD and PAGE experiments strongly suggest that all sequences are able to form monomolecular structures with G-quadruplex domains very similar to the parent TBA. Nevertheless, the type of linker can affect both their physical-chemical and biological properties. Most of the dimeric analogues show thermal stability comparable to TBA, taking into account the experimental error. However, **AA** shows a slightly higher T_m_ than TBA, while **TGlyT** is characterized by a lower T_m_ than TBA. Concerning the affinity to thrombin evaluated by ITC, **AA**, **TT** and **AGlyA** are characterized by dissociation constants with the same order of magnitude as TBA. In contrast, the dissociation constant values for **Gly** and **TGlyT** are higher than TBA, thus showing that these aptamers have a lower affinity to thrombin. Importantly, in the case of RA-36, the ITC measurement has indicated an affinity to thrombin far lower than those showed by derivatives **AA**, **TT** and **AGlyA**. The biological properties of the TBA analogues at low concentration are quite in agreement with the affinity constants to thrombin, with **AA**, **TT** and **AGlyA** showing anticoagulant activities higher than TBA, although a negative effect of the concentration has been highlighted. Interestingly, **AA** and **TT** have shown also the highest stabilities after 6 h in fetal bovine serum, where TBA, RA-36 and the TBA derivatives containing the glycerol moiety have been completely or partially degraded. Although the clinical development of TBA (or HD1) as an anticoagulant drug has been stopped, research on TBA variants with better properties interacting with EXO I is still active, considering the major role of this site in the thrombin inhibition^24^. The whole of our results clearly suggest that the type of dimerization we propose is a useful strategy to improve TBA properties.

## Supplementary information

supplementary information
