# Supplementary material for: Investigating the properties of TBA variants with twin thrombin binding domains
Source: Sci Rep. 2019 Jun 24;9:9184. doi: 10.1038/s41598-019-45526-z (PMC6591170; doi:10.1038/s41598-019-45526-z)
Supplement: Supplementary file 1 — supplementary information [file 41598_2019_45526_MOESM1_ESM.pdf]

SUPPLEMENTARY INFORMATION

INVESTIGATING THE PROPERTIES OF TBA VARIANTS WITH TWIN THROMBIN BINDING DOMAINS

Teresa Amato[1a], Antonella Virgilio[1a], Luciano Pirone[2], Valentina Vellecco[1], Mariarosaria Bucci[1], Emilia Pedone[2], Veronica Esposito[1]* and Aldo Galeone[1]*

***corresponding authors: galeone@unina.it; verespos@unina.it

[1] Dipartimento di Farmacia, Università degli Studi di Napoli Federico II, Via D. Montesano 49, 80131 Napoli, Italy.

[2] Istituto di Biostrutture e Bioimmagini, CNR, Via Mezzocannone 16, 80134, Napoli, Italy

[a] These authors contributed equally.

Synthesis of the non-nucleosidic phosphoramidite building block based on a glycerol residue (Figure S1).

*Compound **1**.*

Propane-1,2,3-triol (glycerol, 220 mg, 2.3 mmol), 4,4-dimethoxytrityl chloride (1.4 g, 4.3 mmol) and 4-dimethylaminopyridine (15 mg, 0.11 mmol) were dissolved in dry pyridine (4 ml). The resulting solution was stirred at room temperature (r.t.) for 3 h. Dry methanol (1 ml) was then added to quench the reaction. After 30 min under stirring, the solution was concentrated under reduced pressure and the residue purified by chromatography on silica gel (eluted with *n*-hexane/ethyl acetate 9:1 v/v) to give compound **1** as a white solid (60% yield; Rf 0.5 in *n*-hexane/ ethyl acetate 8:2 v/v).

*Compound **2**.*

1 (250 mg, 0.36 mmol) was coevaporated with anhydrous diethyl ether and dried *in vacuo* overnight before being dissolved in dry dichlorometane (1 ml) and triethylamine (150 μl, 1.1 mmol) under argon. 96 μl (0.43 mmol) of β-cyanoethyl diisopropylchlorophosphoramidite was then added. After 2 h under stirring at r.t., the reaction mixture was diluted with ethyl acetate (15 ml), and finally washed with 10% sodium carbonate solution (15 ml x 3) and brine (15 ml). The organic layer was dried on anhydrous sodium sulphate and concentrated *in vacuo*. The residue was purified by silica gel chromatography using *n*-hexane/ethyl acetate/triethylamine (8:1:1 v/v/v). The fractions containing the product were collected and concentrated under vacuum, yielding **2** as white foam (90% yield; Rf 0.8 in *n*-hexane/ethyl acetate 7:3 v/v).

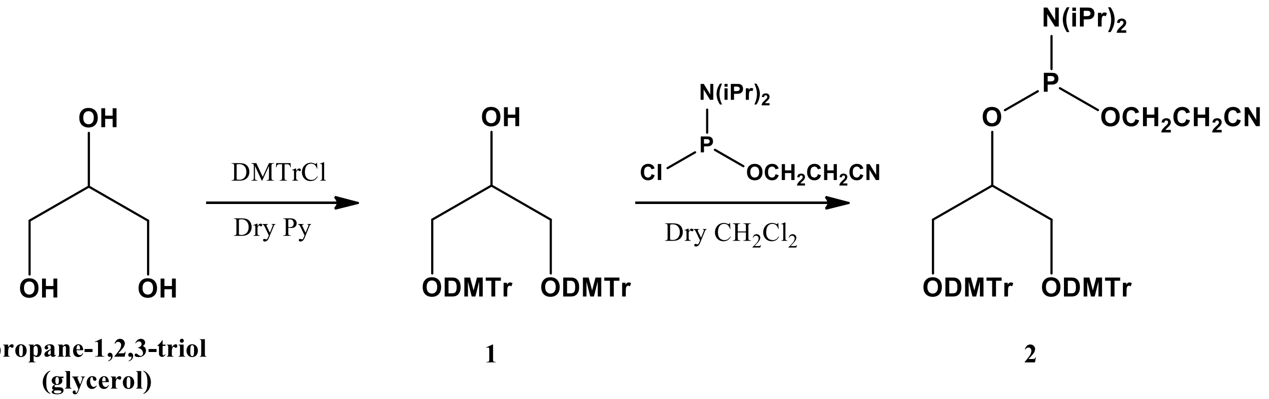

Figure S1: Scheme of the synthetic strategy used to prepare the glycerol linker building block. DMTrCl = 4,4-dimethoxytrityl chloride; iPr = isopropyl.

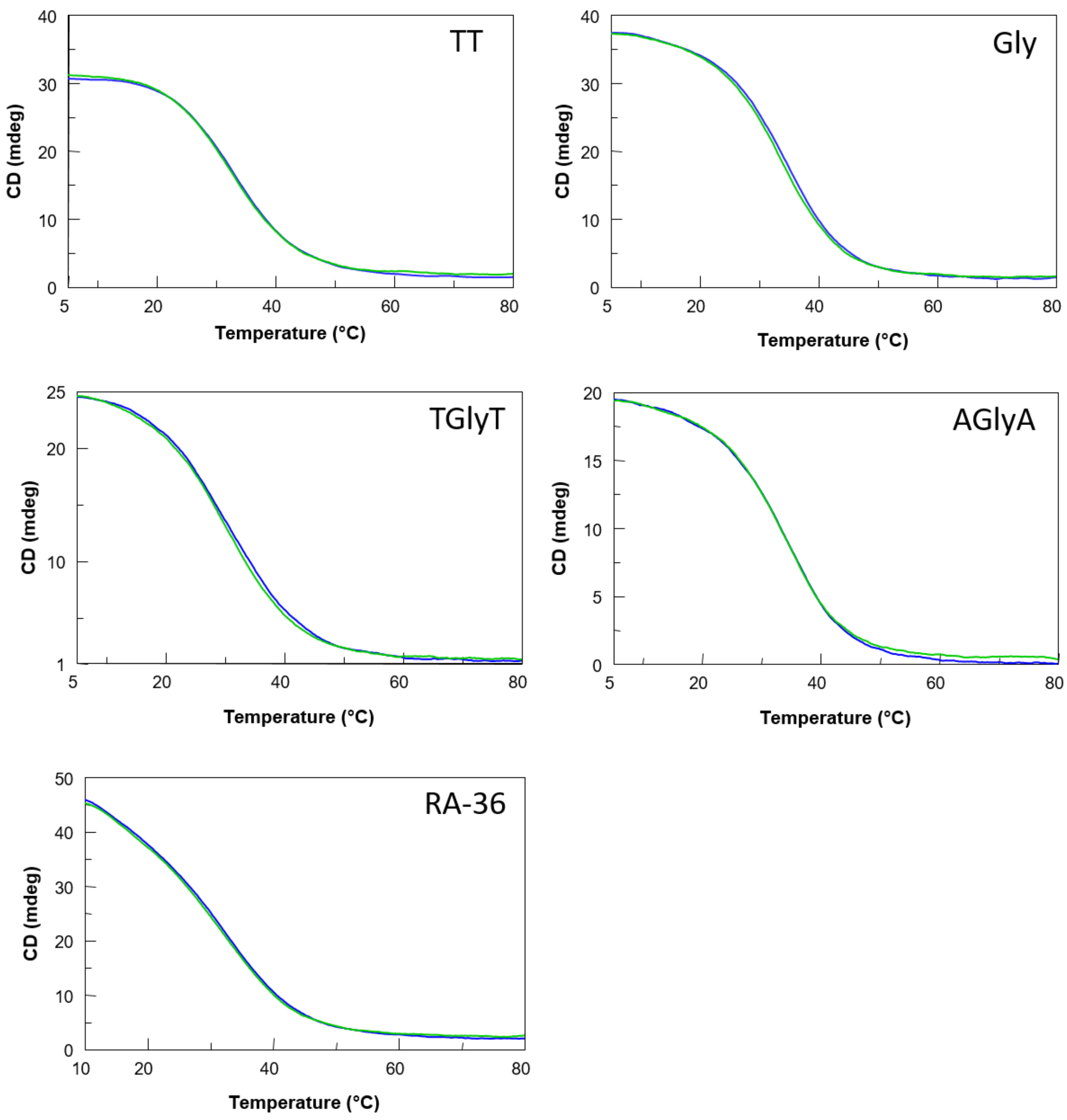

Figure S2. CD melting (blue) and annealing (green) profiles of the G-quadruplex formed by **TT**, **Gly**, **TGlyT**, **AGlyA** and RA-36. See the main text and the experimental section for details.

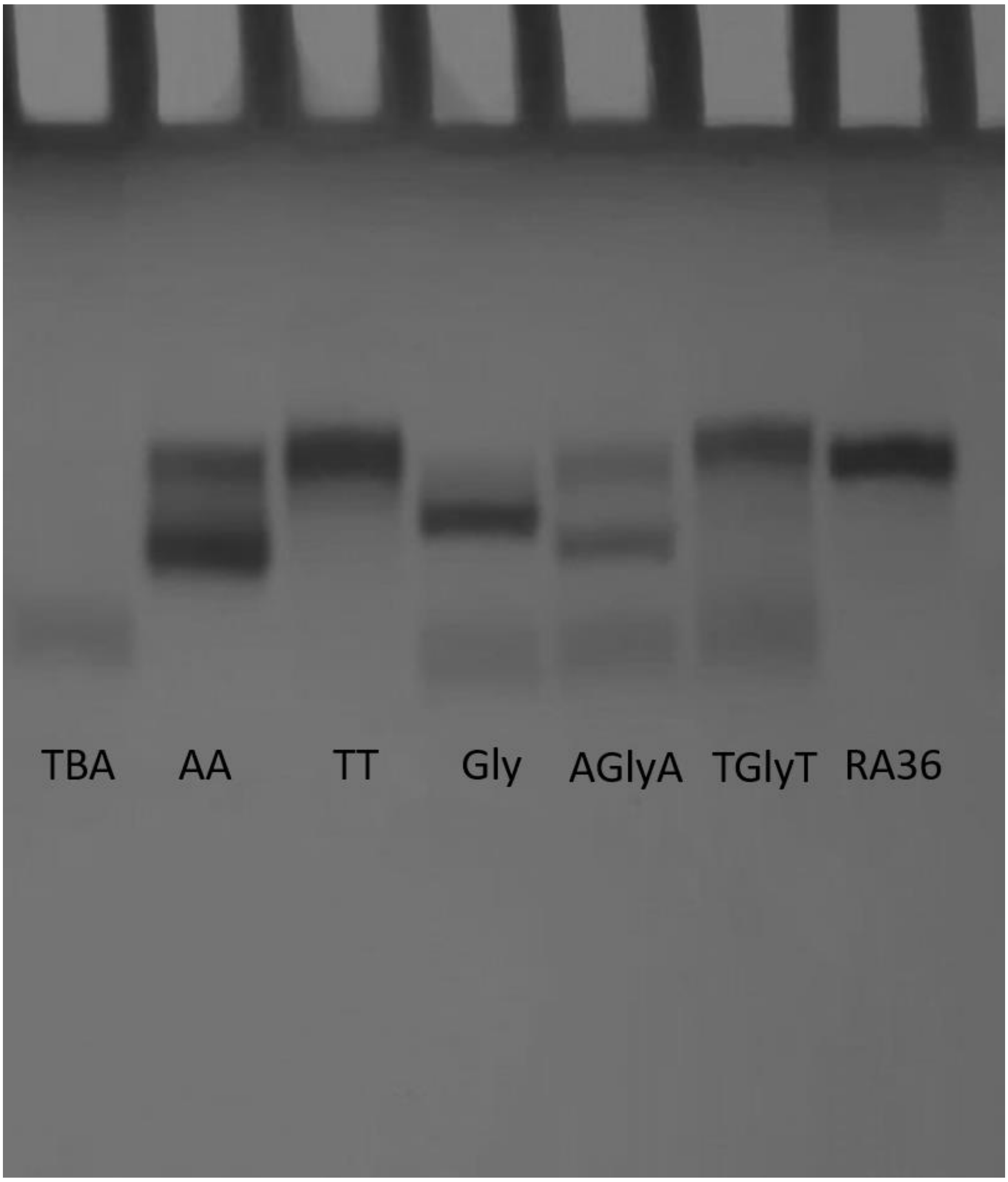

Figure S3. Native polyacrylamide gel electrophoresis of the ODNs investigated (Table 1). G-quadruplex structures of TBA and RA-36 have been used as references. See the main text and the experimental section for details.

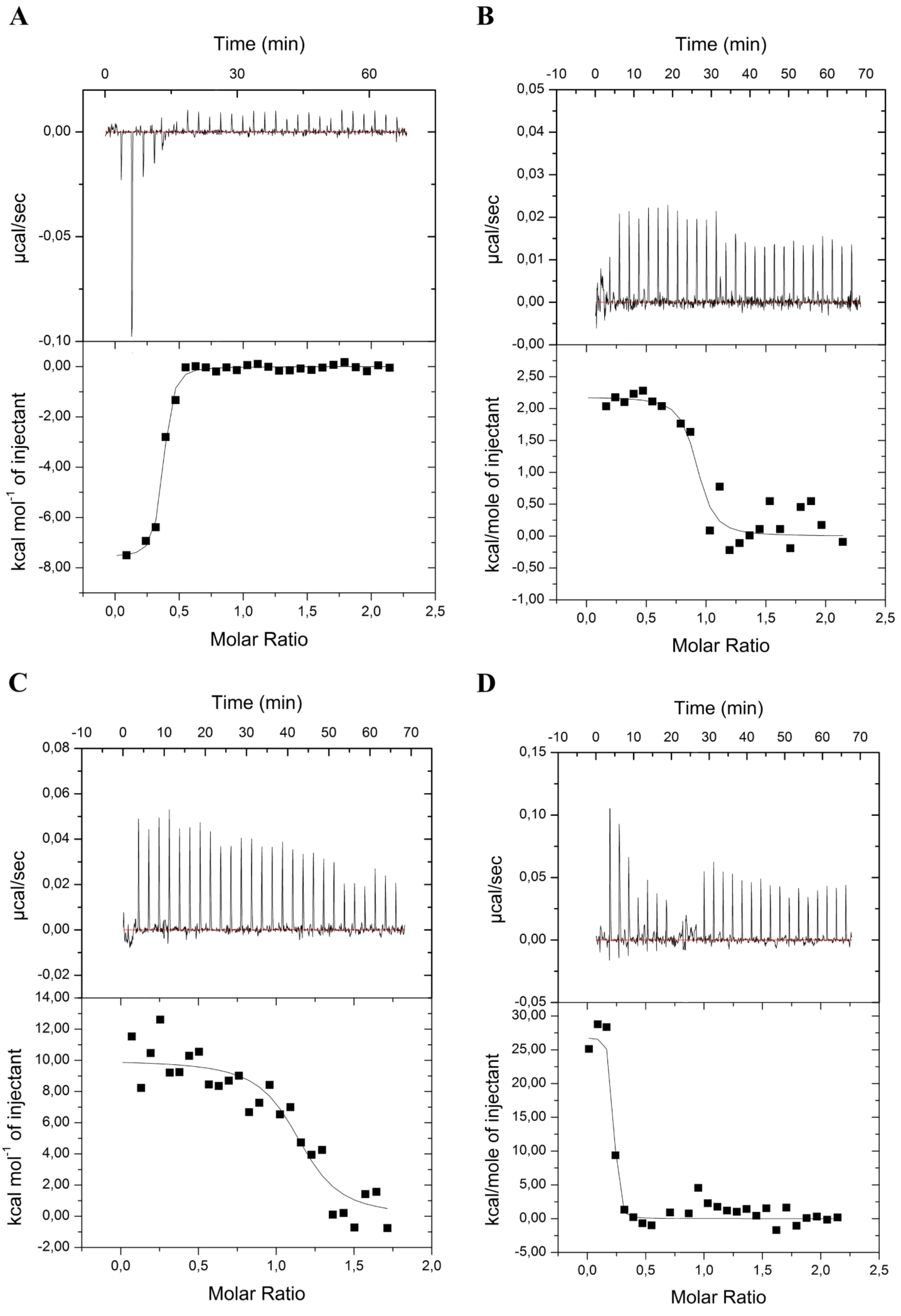

Figure S4: ITC analysis of thrombin binding to different dimeric TBA derivatives. Isothermal titration calorimetry data of thrombin interaction with **AA** (A), **Gly** (B), **TGlyT** (C) and **AGlyA** (D) are shown. The top and bottom panels report raw and integrated data, respectively.

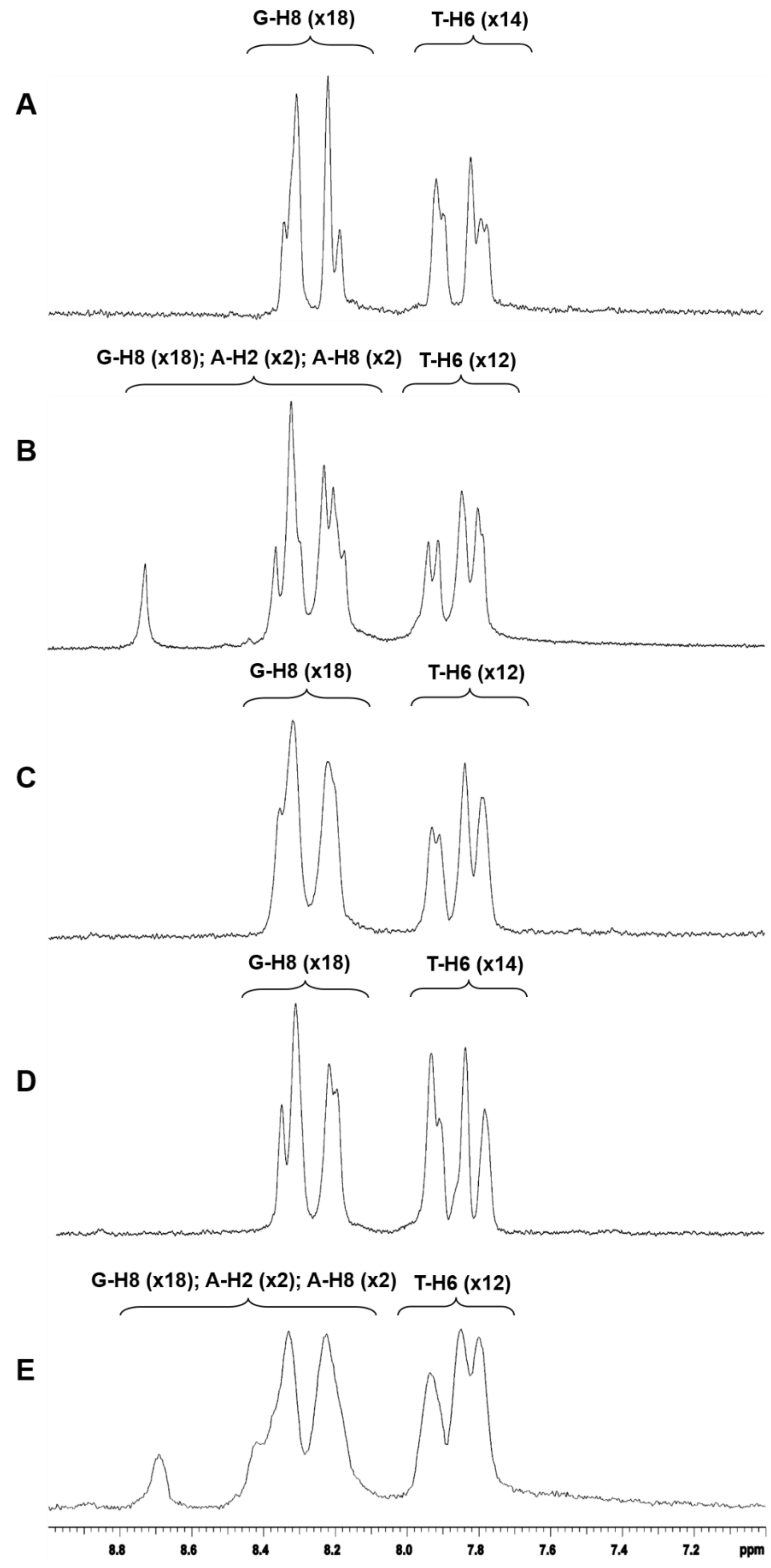

Figure S5. Aromatic region of the NMR spectra (500 MHz, D_2O, 80°C, no salt) of **TT** (A), **AA** (B), **Gly** (C), **TGlyT** (D), **AGlyA** (E). The residual water signal suppression was achieved by presaturation pulse sequence (presat), using a saturation delay of 1.5 sec, a number of scans of 64, a spectral width of 7000 Hz and a number of points of 21000.

Name	Sequence	K_d
TBA	5'-GGTTGGTGTGGTTGG-3'	8 ± 1 nM
**RA-36**	5'-TBA-T-TBA-3'	100 ± 5 nM
**AA**	5'-TBA-A-3'-3'-A-TBA-5'	10 ± 2 nM
**TT**	5'-TBA-T-3'-3'-T-TBA-5'	5 ± 0.5 nM
**Gly**	5'-TBA-3'-Gly-3'-TBA-5'	15 ± 3 nM
**AGlyA**	5'-TBA-A-3'-Gly-3'-A-TBA-5'	5 ± 1 nM
**TGlyT**	5'-TBA-T-3'-Gly-3'-T-TBA-5'	50 ± 7 nM

Table S1: Dissociation constant values (K_d) derived from ITC experiments. See the main text for details.
